# Circular-by-Design Zwitterionic Polyglycerol–Polyoxazoline–Lysine Pseudodendrimers as Multifunctional Antibacterial and Anticancer Drug Delivery Platforms

**DOI:** 10.1021/acs.biomac.6c00382

**Published:** 2026-05-07

**Authors:** Behnaz Bastami, Parviz Rashidi Ranjbar, Mohsen Adeli, Siamak Beyranvand

**Affiliations:** † Kish International Campus, 170457 University of Tehran, Kish Island 39982-79416, Iran; ‡ School of Chemistry, College of Science, University of Tehran, Tehran 141556455, Iran; § Department of Organic Chemistry, Faculty of Chemistry, 117567 Lorestan University, Khorramabad 68141-54316, Iran; ∥ Institut für Chemie und Biochemie, Freie Universität Berlin, Takustr. 3, 14195 Berlin, Germany

## Abstract

The development of multifunctional polymer therapeutics aligned with circular chemistry principles requires renewable building blocks, efficient synthesis, and enhanced material longevity. Herein, we report a circular-by-design zwitterionic pseudodendrimer based on hyperbranched polyglycerol–polyoxazoline–lysine (hPG-*b*-POX-Lys) that integrates antimicrobial activity with targeted anticancer drug delivery. Hyperbranched polyglycerol, derived from glycidol feedstocks, was selectively mesylated and used as a macroinitiator for ring-opening polymerization of 2-ethyl-2-oxazoline, followed by lysine functionalization. The modular synthesis afforded the polymer in 76% yield with high functional density and minimal steps. The zwitterionic architecture exhibited intrinsic antibacterial activity against *Staphylococcus aureus* and *Escherichia coli* with low cytotoxicity. The nanostructure achieved high loading capacity (81.25%) for the CDK4/6 inhibitor palbociclib and enabled efficient in vitro delivery, improving selectivity toward cancer cells. This platform demonstrates a scalable strategy for multifunctional, circular nanomedicine design.

## Graphical abstract

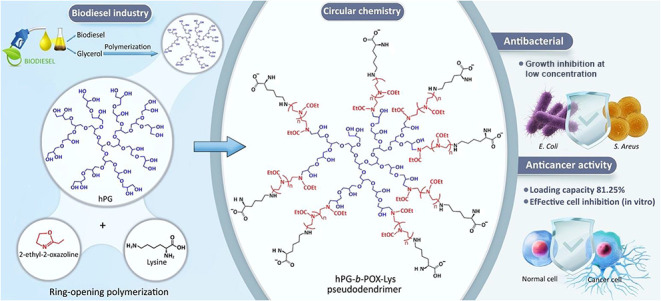

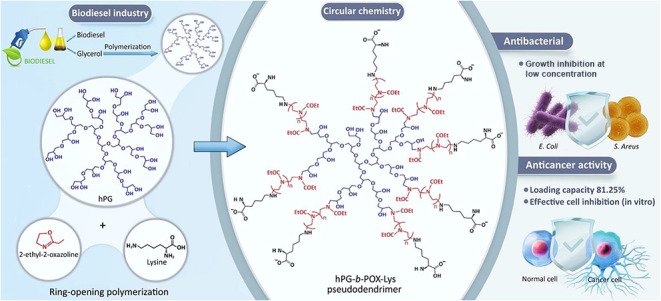

## Introduction

The rapid emergence of antibiotic-resistant bacteria represents a critical global health challenge, demanding therapeutic strategies that move beyond conventional small-molecule antibiotics.^1^ Conventional antibiotics are increasingly ineffective due to the rapid adaptation of pathogenic bacteria and the resistance mechanisms they develop. The accelerated evolution of resistance mechanisms has rendered many frontline antibiotics ineffective, highlighting the urgent need for antimicrobial systems that operate through alternative physicochemical modes of action while minimizing resistance development.^2^ From a circular chemistry perspective, the design of multifunctional polymeric systems that reduce material redundancy and extend therapeutic performance is particularly attractive, as such systems can integrate multiple biological functions within a single, modular architecture.^3–5^

Concurrently, cancer continues to impose a substantial global health burden, necessitating drug delivery platforms that enhance therapeutic selectivity while reducing systemic toxicity.^6,7^ Polymeric materials^8^ have emerged as promising candidates for both antimicrobial and anticancer applications due to their structural tunability,^9^ functional group density, and capacity for nanoscale organization.^10,11^ Among these, high functional polymers^12–14^ are especially compelling because their highly branched architectures enable multivalent interactions with bacterial membranes and cancer cells, facilitating membrane disruption, enhanced cellular uptake, and selective cytotoxicity.^15–19^ Designing such macromolecular systems using biocompatible and potentially bioderived building blocks further supports circular materials innovation in nanomedicine.^20,21^

Hyperbranched polyglycerol (hPG) represents an attractive core scaffold for pseudodendrimers^22^ due to its biocompatibility,^23–25^ low toxicity, and ease of functionalization.^26^ Importantly, polyglycerol can be synthesized from glycidol-based feedstocks, offering compatibility with renewable resource streams and step-economical polymerization strategies. When combined with polyoxazoline (POX),^27,28^ a stealth polymer known for its biocompatibility and protein-repellent properties, the resulting architecture benefits from prolonged circulation potential and reduced nonspecific interactions.^29,30^ Functionalization with lysine,^31^ a naturally occurring amino acid, introduces biodegradable linkages and intrinsic antimicrobial character,^32–34^ enhancing membrane interactions while maintaining biocompatibility.^35–39^

From a Circular-by-Design perspective, the hPG-*b*-POX-Lys architecture is intentionally engineered to reduce material redundancy by integrating multiple therapeutic functions within a single macromolecular platform. Instead of employing separate materials for antimicrobial protection, drug delivery, and biocompatibility enhancement, the present pseudodendrimer combines these features through its modular design. The hyperbranched polyglycerol core provides a biocompatible scaffold, the polyoxazoline segment contributes stealth and drug-loading capability, and lysine termini introduce intrinsic antimicrobial activity and biodegradable functionality. This multifunctional integration enables a single material system to replace several independent components, thereby simplifying formulation design, improving therapeutic efficiency, and aligning the material development strategy with circular chemistry principles.

In this work, we report the synthesis and characterization of a zwitterionic hPG-*b*-POX-Lys pseudodendrimer engineered as a dual-function therapeutic platform. The material exhibited measurable antibacterial activity, with minimum inhibitory and bactericidal concentrations of 190 μg/mL against *Staphylococcus aureus* and *Escherichia coli*, demonstrating effective broad-spectrum activity. Simultaneously, the nanocarrier showed high loading capacity (81.25%) for the CDK4/6 inhibitor Palbociclib and displayed pH-responsive release behavior, achieving 84.2% drug release at pH 5.5, indicative of accelerated release under acidic tumor-mimicking conditions. At 30 μg/mL (hPG-*b*-POX-Lys+Palbociclib), the formulation induced a pronounced synergistic anticancer effect in breast cancer cells while maintaining favorable biocompatibility.

By integrating renewable-compatible scaffolds, amino acid functionalization, intrinsic antibacterial activity, and targeted anticancer drug delivery within a single macromolecular construct, this study advances the development of multifunctional biomacromolecules aligned with circular chemistry principles. The presented pseudodendrimer platform illustrates how rational macromolecular engineering can contribute to more sustainable and therapeutically efficient materials for next-generation biomedical applications.

## Experimental Section

Materials, methods and some of characterizations can be found in Electronic Supporting Information (SI).

### Synthesis of Mesylated Hyperbranched Polyglycerol (hPG-OMS)

Hyperbranched polyglycerols (hPG; *M_n_* = 5000 Da), (1 mmol, 5 g) were first dried under vacuum at 50 °C. Under an inert argon atmosphere, the dried hPG was dissolved in pyridine (50 mL) at 0 °C. Mesyl chloride (5.5 mL, 71 mmol) was separately dissolved in pyridine at 0 °C and then added dropwise to the hPG solution over 1 h. The reaction mixture was subsequently stirred at room temperature (500 rpm) under argon for 22 h. The crude product was purified by dialysis against acetone for 24 h. The reaction yielded approximately 50%, and the resulting hPG-OMS was characterized by FT-IR and NMR spectroscopy. The density of mesyl groups was also 50%.

### Synthesis of Poly­(glycerol-*b*-2-ethyl-2-oxazoline)-lysine (hPG-*b*-POX-Lys)

Mesylated hyperbranched polyglycerol (hPG-OMS, 500 mg) was dissolved in dry acetonitrile (final volume 200 mL) in a three-neck round-bottom flask under an argon atmosphere. The reaction mixture was heated to 80 °C using an oil bath under reflux conditions. Subsequently, 2-ethyl-2-oxazoline (5 mL, 50 mmol) was added, and the mixture was stirred at 500 rpm for 48 h.

The polymerization was quenched by the addition of lysine. In this step, 500 mg of lysine was dissolved in a distilled water/acetonitrile mixture (1:10 v/v). Water was partially removed using a rotary evaporator at elevated temperature, and the resulting solution was added to the reaction flask. The mixture was stirred overnight at 80 °C.

After cooling, the mixture was filtered through paper and dialyzed in a two-step process: first for 24 h against saturated distilled water/sodium chloride solution using a 2000 Da cutoff dialysis membrane, followed by 48 h of dialysis in distilled water. The final product, obtained as a cream-colored powder, was dried at 45 °C and characterized by NMR and IR spectroscopy.

### Zone of Inhibition Assay (Well Diffusion Method)

The antimicrobial activity of the samples was evaluated using the well diffusion method by measuring the zones of inhibition against bacterial growth. Two standard bacterial strains were employed: the Gram-positive *S. aureus* (ATCC 25923) and the Gram-negative *E. coli* (ATCC 25922).

Bacterial suspensions were prepared according to the 0.5 McFarland standard (≈1.5 × 10^8^ CFU/mL) using phosphate-buffered saline, followed by serial dilution to the desired concentration. The inoculum was uniformly spread across Mueller-Hinton agar plates using a sterile cotton swab. Sterile wells (5 mm diameter) were aseptically punched into the agar using a stainless-steel cylinder, maintaining sufficient spacing to prevent overlapping zones.

Test samples (50–80 μL of hPG, hPG-*b*-POX-Lys, or chloramphenicol as a positive control) were added to the wells using a sterile micropipette. Plates were preincubated at 4 °C for 2 h to facilitate diffusion, followed by incubation at 37 °C. Zones of inhibition were measured after the incubation period. All experiments were performed in triplicate to ensure reproducibility.

### Cell Viability Assessment Using the MTT Assay

MCF-7, HeLa, and human fibroblast cells were seeded into 96-well plates at a density of 1 × 10^4^ cells per well and incubated at 37 °C with 5% CO_2_ for 24 h. Cells were then treated with Palbociclib and hPG-*b*-POX-Lys+Pal. at low concentrations to determine IC_50_ values accurately. Higher concentrations of hPG-*b*-POX-Lys alone were applied to evaluate its intrinsic cytotoxicity. All treatments were performed for 48 h.

Following treatment, 20 μL of MTT solution (5 mM) was added to each well and incubated for 4 h in the dark to allow for formazan crystal formation. The formazan crystals were solubilized in 100 μL of DMSO, and absorbance was measured at 570 nm with a reference wavelength of 630 nm.

All experiments were conducted in triplicate and independently repeated three times. Data are expressed as mean ± SD and analyzed using one-way ANOVA followed by Tukey’s post hoc test, with statistical significance set at *P* < 0.05.

### Preparation of Palbociclib-Loaded hPG-*b*-POX-Lys (hPG-*b*-POX-Lys+Pal.)

To enhance the therapeutic potential of hPG-*b*-POX-Lys, Palbociclib, a potent CDK4/6 inhibitor, was selected as the model drug for encapsulation. For drug loading, 0.15 mg of Palbociclib and 0.03 mg of the synthesized polymer were dissolved in 3 mL of phosphate-buffered saline (PBS, pH 7.4) and stirred continuously at room temperature for 24 h to ensure optimal drug-polymer interaction.

After incubation, the solution was centrifuged at 6000 rpm to remove unbound aggregates, and the supernatant was transferred into a dialysis membrane. Dialysis was performed against 1 L of deionized water–ethanol solution (50:50, v/v) for 5 h, with the solution refreshed every hour. This step removed residual solvents and unencapsulated drug, enhancing the purity and stability of the drug-loaded polymer. The final product was obtained by solvent evaporation followed by vacuum drying. The high loading capacity (81.25%) confirms efficient encapsulation within the pseudodendrimeric architecture of hPG-*b*-POX-Lys.

### Determination of Minimum Inhibitory Concentration (MIC) and Minimum Bactericidal Concentration (MBC)

The MIC of the dendrimer formulations was determined using the broth microdilution method according to CLSI guidelines. Bacterial suspensions were adjusted to 0.5 ± 0.02 McFarland units (∼1 × 10^8^ CFU/mL) and serial 2-fold dilutions of the dendrimer samples were prepared in sterile 96-well round-bottom microplates (Thermo Fisher Scientific, Roskilde, Denmark) across the desired concentration range. Each well was inoculated to a final bacterial density of 1 × 10^6^ CFU/mL and incubated statically at 37 °C under 5% CO_2_ for 24 h. The MIC was recorded as the lowest concentration at which no visible turbidity was observed, indicating complete inhibition of bacterial growth.

The MBC was subsequently determined using a flash microbicidal assay. Briefly, 180 μL of tryptose soy broth (TSB) and 20 μL of inoculum from the MIC wells were transferred into 96-well flat-bottom microplates and incubated under the same conditions. The MBC was defined as the lowest concentration of dendrimer that completely eradicated bacterial viability, confirmed by the absence of visible growth. All assays were performed in quadruplicate (*n* = 4) to ensure reproducibility. Data are expressed as mean ± SD and analyzed using one-way ANOVA followed by Tukey’s post hoc test, with statistical significance set at *P* < 0.05.

## Results and Discussion

The hPG-*b*-POX-Lys pseudodendrimer was successfully synthesized via a multistep route, as outlined in Scheme 1.^40^ Briefly, hyperbranched polyglycerol (hPG) was mesylated (hPG-OMS) and employed as a macroinitiator for the ring-opening polymerization of 2-ethyl-2-oxazoline,^41^ yielding hPG-*b*-POX, followed by lysine termination. Structural characterization confirmed the successful formation of the targeted pseudodendrimer architecture (hPG-*b*-POX-Lys).

**Scheme 1:**
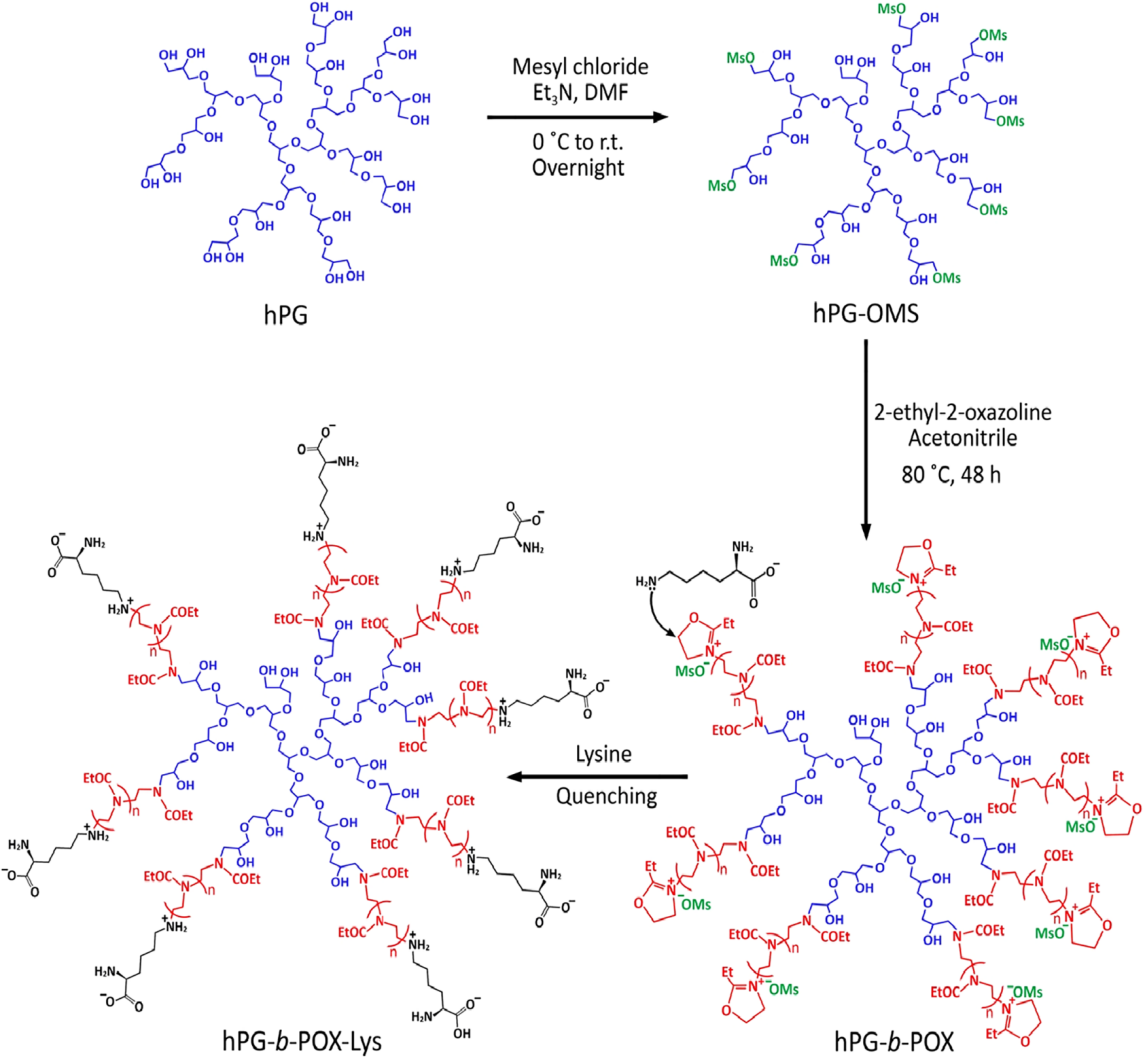
Schematic Representation of the Synthesis of hPG-*b*-POX-Lys^a^

a Hyperbranched polyglycerol was mesylated and used as a macroinitiator for the ring opening polymerization of 2-ethyl-2-oxazoline. Termination of polymerization by lysine resulted in hPG-*b*-POX-Lys.

The successful stepwise synthesis of hPG-*b*-POX-Lys was confirmed by Fourier-transform infrared (FT-IR) spectroscopy (Figure 1a). The FT-IR spectrum of hyperbranched polyglycerol (hPG) (Figure 1ai) exhibits a broad O–H stretching band centered around 3300 cm^–1^, characteristic C–H stretching vibrations in the range of 2890–2980 cm^–1^, and strong C–O stretching bands between 1000 and 1300 cm^–1^, which are consistent with reported spectra of hPG.

**Figure 1:**
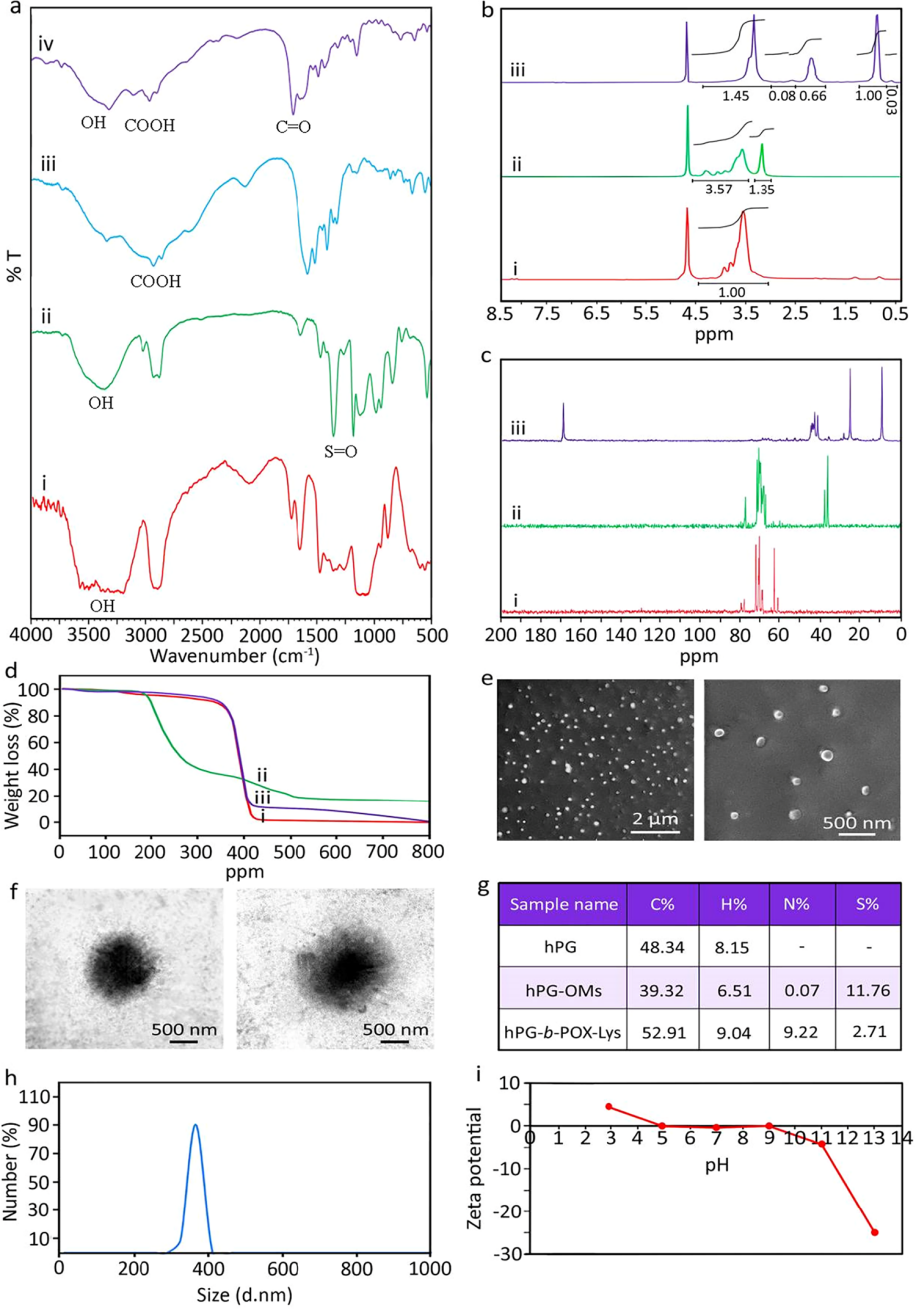
(a) IR spectra of (i) hPG, (ii) hPG-OMS, (iii) Lys and (iv) hPG-*b*-POX-Lys. (b) ^1^H NMR and (c) ^13^C NMR spectra of (i) hPG, (ii) hPG-OMS, and (iii) hPG-*b*-POX-Lys. (d) TGA thermograms of (i) hPG, (ii) hPG-OMS, Lys and (iii) hPG-*b*-POX-Lys. (e) SEM and (f) TEM images of hPG-*b*-POX-Lys. (g) CHNS analysis for hPG, hPG-OMS and hPG-*b*-POX-Lys. (h) DLS diagram and (i) zeta potential of hPG-*b*-POX-Lys at different pHs.

Following mesylation, the FT-IR spectrum of hPG-OMS (Figure 1a­(ii)) shows a noticeable reduction in the intensity of the O–H stretching band at approximately 3300 cm^–1^, indicating the consumption of hydroxyl groups. In addition, a characteristic S=O stretching vibration appears at around 1350 cm^–1^, together with C–H stretching bands near 2800–2900 cm^–1^, confirming the successful introduction of mesylate groups onto the hPG backbone.

The FTIR spectrum of lysine shows the characteristic vibrational bands associated with amino acid functional group (Figure 1a­(iii)). A broad absorption band in the 3200–3500 cm^–1^ region is observed, corresponding to overlapping N–H and O–H stretching vibrations from the amino and hydroxyl groups involved in hydrogen bonding. The bands near 2930–2850 cm^–1^ are attributed to aliphatic C–H stretching vibrations of the methylene groups in the lysine side chain. A strong band in the region around 1600–1650 cm^–1^ is assigned to the asymmetric stretching vibration of the carboxylate (COO^–^) group, while the band near 1400–1450 cm^–1^ corresponds to its symmetric stretching mode. The absorption features observed around 1500–1550 cm^–1^ are associated with N–H bending vibrations of the protonated amino groups.

In the FT-IR spectrum of the final hPG-*b*-POX-Lys pseudodendrimer (Figure 1a­(iv)), the presence of lysine is confirmed by the appearance of characteristic amide bands, including an amide I C=O stretching vibration near 1650 cm^–1^ and an amide II N–H bending band in the range of 1550–1570 cm^–1^. The further intensification of the broad band in the 3200–3600 cm^–1^ region is consistent with the increased contribution of N–H groups from lysine residues. These spectral features collectively verify the successful termination of the polymerization with lysine and the formation of the targeted hPG-*b*-POX-Lys architecture.

Nuclear magnetic resonance (NMR) spectroscopy was employed to confirm the chemical structure and successful stepwise functionalization of the synthesized pseudodendrimers (Figure 1b and c).

In the ^1^H NMR spectrum of hyperbranched polyglycerol (hPG) (Figure 1b­(i)), the characteristic proton resonances of the polyglycerol backbone appear as broad signals in the range of 3.5–4.5 ppm, corresponding to methine and methylene protons adjacent to hydroxyl groups, in agreement with reported spectra of hPG.

Following mesylation, the ^1^H NMR spectrum of hPG-OMS (Figure 1b­(ii)) displays a distinct singlet at approximately 3.2 ppm, assigned to the methyl protons of the mesyl group (CH_3_–SO_2_−), alongside the backbone polyglycerol signals at 3.4–3.8 ppm. Integration of these resonances indicates that approximately 50% of the hydroxyl groups were converted to mesylate functionalities, confirming successful partial mesylation of hPG.

The ^1^H NMR spectrum of the final hPG-*b*-POX-Lys pseudodendrimer (Figure 1b­(iii)) shows additional signals arising from the polyoxazoline and lysine segments. Resonances at 0.87 and 2.17 ppm are attributed to the terminal methyl and methylene protons of the ethyl side chains (CH_3_–CH_2_−) of the poly­(2-ethyl-2-oxazoline) block. Weak signals observed at 1.0–1.3 ppm correspond to methylene protons of the lysine side chain (−CH_2_–CH_2_–CH_2_−), while the signal at approximately 3.27 ppm is assigned to the – N–CH_2_– protons of lysine. Broad resonances at around 3.5 and 3.6 ppm are attributed to overlapping signals from the polyoxazoline backbone and the hyperbranched polyglycerol core, respectively (Figure S1a).

The ^13^C NMR spectrum of hPG (Figure 1c­(i)) exhibits characteristic signals in the range of 60–85 ppm, corresponding to methylene and methine carbons of the polyglycerol backbone. After mesylation, the ^13^C NMR spectrum of hPG-OMS (Figure 1c­(ii)) shows two distinct signals at 36.6 and 38.2 ppm, attributed to the methyl carbons of mesyl groups located in different chemical environments, further confirming successful mesylation.

In the ^13^C NMR spectrum of hPG-*b*-POX-Lys (Figure 1c­(iii)), signals corresponding to the polyoxazoline segments are clearly observed, including resonances at approximately 8 and 26 ppm assigned to the ethyl side-chain carbons (−CO–CH_2_CH_3_), signals in the range of 43–48 ppm attributed to −N–CH_2_–CH_2_–N– carbons of the polyoxazoline backbone, and carbonyl carbon signals at 177.4 and 177.6 ppm. An additional resonance at around 178 ppm is assigned to the carbonyl carbon of the lysine moiety (Figure S1b). Further lysine-related signals appear at 8.5, 9.5, and 29 ppm for methylene carbons of the side chain, as well as at approximately 37 ppm (−N–CH–COO^–^) and 46 ppm (−N–CH_2_−). Weak signals corresponding to the polyglycerol core remain visible in the range of 50–75 ppm due to signal overlap and the branched architecture of the pseudodendrimer.

The thermal stability of hPG-*b*-POX-Lys was evaluated by thermogravimetric analysis (TGA) over a temperature range of 25–800 °C (Figure 1d). The TGA profile of hPG shows a major weight-loss at 420 °C, which is attributed to the thermal degradation of the polyglycerol backbone, in agreement with previously reported data for hyperbranched polyglycerol. Also, higher than 100 °C and lower than main degradation a ∼8% weight loss is assigned to the adsorbed water in hPG.

Figure 1d­(ii) presents the TGA thermogram of mesylated hPG (hPG-OMS). Compared with pristine hPG, an additional weight-loss step is observed in the temperature range of 210–300 °C, which can be attributed to the thermal cleavage of mesylate groups. This weight loss then broadens and continues up to approximately 510 °C, corresponding to the decomposition of the hPG backbone. The relative magnitude of the mass loss associated with the mesyl groups is consistent with the partial mesylation of hPG, as independently estimated by ^1^H NMR analysis.

The TGA thermogram of the final hPG-*b*-POX-Lys pseudodendrimer is shown in Figure 1d­(iii). Its thermal profile closely resembles that of hPG, while also reflecting the characteristic behavior of polyoxazoline. The primary weight loss, attributed to the decomposition of the copolymer backbone, occurs at approximately 420 °C.

Scanning Electron Microscopy (SEM) revealed spherical polymeric particles with diameters in the range of 300–400 nm (Figures 1e and S2). The particles exhibited a relatively narrow size distribution, indicating a uniform morphology. Transmission Electron Microscopy (TEM) images further supported these observations, showing spherical particles consistent with the SEM images and DLS results (Figures 1f,h and S3). Although TEM imaging was challenging due to the limited stability of the polymeric particles under the electron beam, numerous well-defined spherical particles were clearly observed.

Differential scanning calorimetry (DSC) was employed to evaluate the thermal transition behavior of the synthesized polymers. As shown in Figure S4, the glass transition temperature (*T*_g_) increased from – 25 °C for pristine hPG to 47 °C for hPG-*b*-POX-Lys. This pronounced shift in Tg reflects the incorporation of poly­(2-ethyl-2-oxazoline) and lysine segments into the polymer framework and is consistent with the successful formation of the modified pseudodendrimer structure.

The elemental composition of hPG-*b*-POX-Lys was thoroughly characterized by Energy Dispersive X-ray Spectroscopy (EDX) (Figure S5). EDX spectra of unmodified hPG showed only carbon and oxygen, confirming its pristine polyol structure. Upon mesylation, hPG-OMS exhibited a notable decrease in carbon content along with 9.23% sulfur, indicating efficient introduction of mesyl groups. Subsequent polymerization of oxazoline resulted in a substantial increase in nitrogen content (7.92%) and a concurrent reduction of sulfur to 0.47%, consistent with the formation of the hPG-*b*-POX-Lys block copolymer. These findings were further validated by CHNS elemental analysis (Figure 1g), which confirmed the successful oxazoline polymerization initiated by hPG-OMS. Based on the sulfur content of hPG-*b*-POX-Lys, the density of lysine functionalities in the copolymer was estimated to be approximately 38%.

A detailed comparison of the elemental compositions revealed that mesylation decreased carbon (39.32%) and hydrogen (6.51%) contents, while sulfur content increased to 11.76%, reflecting the incorporation of mesyl groups. Following oxazoline polymerization and lysine termination, sulfur content markedly decreased, whereas nitrogen, carbon, and hydrogen contents increased, demonstrating effective replacement of mesyl groups with oxazoline units and incorporation of lysine residues.

These systematic compositional changes provide compelling evidence for the stepwise synthesis of hPG-*b*-POX-Lys and the successful integration of lysine, which introduces nitrogen into the polymer backbone.

The synthesized polymer exhibits zwitterionic characteristics due to the presence of lysine residues containing both carboxylate and ammonium groups. The isoelectric point (pI) of hPG-*b*-POX-Lys was determined to be approximately 5.1, reflecting the pH at which the polymer carries no net charge. Zeta potential measurements confirmed this behavior: the particles are positively charged at acidic pH (<5), negatively charged at alkaline pH (>5.1), and approach neutrality around the isoelectric point (pH ≈ 5.1), consistent with the zwitterionic nature of the polymer (Figure 1i).

The drug loading performance of the hPG-*b*-POX-Lys pseudodendrimer was quantified using two standard parameters: loading capacity (LC) and drug loading efficiency (DLE). LC represents the weight fraction of the drug in the final drug-loaded formulation and was calculated according to Equation X. The LC of the hPG-*b*-POX-Lys+Pal. complex was determined to be 81.25%, indicating a high drug content relative to the total carrier mass (Table S1).

DLE reflects the fraction of the initially added drug that was successfully incorporated into the carrier. It was determined by dissolving the dried complex in PBS and measuring the absorbance at 267 nm using UV–Vis spectroscopy based on a calibration curve (*y* = 0.0176*x* – 0.0882, *R* ^2^ = 0.9966, Figure S6). The DLE value was 86%, confirming efficient drug incorporation during the loading process.

The consistently high LC and DLE values demonstrate the strong loading performance of the hPG-*b*-POX-Lys carrier toward Palbociclib.

The cytotoxic effects of free Palbociclib, the zwitterionic hPG-*b*-POX-Lys pseudodendrimer, and the hPG-*b*-POX-Lys+Pal complex were evaluated in MCF-7 breast cancer cells, HeLa cervical cancer cells, and human fibroblasts using the MTT assay after 48 h of incubation. The IC_50_ values for free Palbociclib and the drug-loaded polymer are shown in Figure 2.

**Figure 2:**
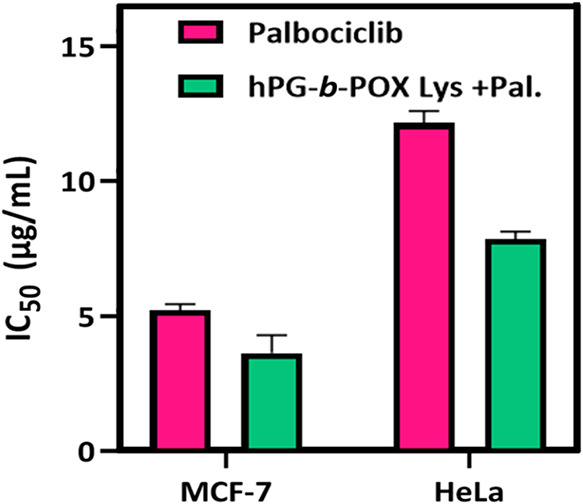
IC_50_ values of free Palbociclib and the hPG-*b*-POX-Lys+Pal. complex in MCF-7 and HeLa cancer cells after 48 h incubation, assessed by the MTT assay. Data are presented as mean ± SD (*n* = 3). The hPG-*b*-POX-Lys+Pal. complex exhibited enhanced cytotoxicity in MCF-7 and HeLa cells compared with free Palbociclib, with IC_50_ values of 3.48 μg/mL (MCF-7) and 7.57 μg/mL (HeLa).

The half-maximal inhibitory concentration (IC_50_) represents the concentration of compound required to reduce cell viability by 50% relative to untreated control. The corresponding IC_50_ values, together with 95% confidence intervals are summarized in Table 1.

**Table 1:** IC_50_ Values are Reported as Mean ± SD (*n* = 3)^a^

Compound	MCF-7 Cell	HeLa Cell	Fibroblasts Cell	IC_50_ Fibroblasts Cell/IC_50_ HeLa Cell
IC_50_ (Palbociclib) (μg/mL)	5.01	11.69	>12	1.02
IC_50_ (hPG-*b*-POX-Lys+Pal.) (μg/mL)	3.48	7.57	>12	1.58
IC_50_ (hPG-*b*-POX-Lys) (μg/mL)	>5000	>5000	>5000	∼1

a In human fibroblasts, hPG-*b*-POX-Lys showed low cytotoxicity, and neither the polymer carrier nor the hPG-*b*-POX-Lys+Pal complex reduced cell viability below 50% within the tested concentration range, indicating favorable cytocompatibility.

Free Palbociclib exhibited pronounced antiproliferative activity in both caf ncer cell lines, with IC_50_ values of 5.01 μg/mL in MCF-7 cells and 11.69 μg/mL in HeLa cells. Incorporation of Palbociclib into the hPG-*b*-POX-Lys pseudodendrimer enhanced its anticancer activity in MCF-7 cells (IC_50_ = 3.48 μg/mL), indicating that polymer conjugation improved the drug’s efficacy, possibly through enhanced cellular uptake or sustained release.

In contrast, the polymer alone produced only a gradual, dose-dependent decrease in cell viability and required substantially higher concentrations to achieve comparable effects, consistent with its intended role as a delivery platform rather than a strongly cytotoxic agent.

In HeLa cells, the hPG-*b*-POX-Lys+Pal complex showed somewhat lower activity compared with MCF-7 cell. Such variations between cell lines are commonly observed and may be attributed to differences in cellular uptake, intracellular trafficking, and drug sensitivity in polymer-based delivery systems indicating enhanced anticancer activity following polymer-mediated delivery.

In human fibroblasts, neither free Palbociclib nor the hPG-*b*-POX-Lys+Pal complex reached an IC_50_ within the investigated concentration range (IC_50_ > 12 μg/mL), suggesting limited cytotoxicity toward nonmalignant cells under the tested conditions. In contrast, the unloaded hPG-*b*-POX-Lys pseudodendrimer showed minimal effects on cell viability, with IC_50_ values exceeding the highest tested concentration (>5000 μg/mL) across all cell lines, confirming its favorable in vitro biocompatibility. Overall, these results demonstrate that incorporation of Palbociclib into the zwitterionic hPG-*b*-POX-Lys pseudodendrimer enhances anticancer efficacy in cancer cells while maintaining low cytotoxicity toward normal fibroblasts, supporting its potential as a safe and effective drug delivery platform.

Palbociclib exhibits very low inhibitory concentrations against its molecular targets CDK4 and CDK6 in biochemical assays, with reported IC_50_ values in the low nanomolar range (≈ 9–16 nM).^42^ However, in cell-based viability assays such as MTT, the effective concentrations required to achieve a 50% reduction in cell proliferation are typically higher and often fall within the micromolar range. This discrepancy reflects the drug’s cytostatic mechanism of action, primarily inducing cell-cycle arrest rather than direct cytotoxicity,^43^ and is consistent with the IC_50_ values reported in Table 1 and previous MTT-based studies.

The conjugation of Palbociclib with hPG-*b*-POX-Lys enhanced its antiproliferative activity in cancer cell lines. The IC_50_ values decreased from 5.013 to 3.48 μg/mL in MCF-7 cells and from 11.69 to 7.57 μg/mL in HeLa cells for the polymer-drug complex compared with the free drug. This improvement may be associated with enhanced cellular uptake and improved intracellular drug availability mediated by the polymeric carrier.

In human fibroblast cells, neither the unloaded polymer nor the hPG-*b*-POX-Lys+Pal complex reduced cell viability below 50% within the tested concentration range. Accordingly, IC_50_ values were not reached under the experimental conditions (IC_50_ > highest tested concentration). Cell viability remained high even at the maximum applied dose, supporting the favorable cytocompatibility profile of the carrier system.

For free Palbociclib, a concentration-dependent reduction in fibroblast viability was observed; however, the IC_50_ threshold was likewise not reached within the tested concentration window. Overall, these findings indicate that polymer-based delivery preserves or improves anticancer activity while maintaining low cytotoxic effects in nonmalignant cells within the examined dose range. The IC_50_ (fibroblast cells)/IC_50_ (HeLa cells) ratio was used as the selectivity index for the different agents. As shown, the selectivity of hPG-*b*-POX-Lys+Pal is higher than that of the free drug, highlighting the improved safety of this compound as a drug delivery system.

The release kinetics of Palbociclib from hPG-*b*-POX-Lys was evaluated at physiological (pH 7.4) and acidic (pH 5.5) conditions to simulate both normal tissue and tumor microenvironments, respectively. For this purpose, 3 mL of the aqueous solution containing the hPG-*b*-POX-Lys+Pal. complex was added in a dialysis membrane (MWCO: 2000 Da) and submerged in 30 mL of phosphate-buffered saline (PBS) maintained at the specified pH conditions. The experimental setup was designed to ensure sink conditions by replacing the external buffer at predefined intervals, which allowed the sustained removal of released drug molecules and prevented saturation.

The release profile of Palbociclib was quantitatively monitored by measuring the UV absorbance of the dialysate at its characteristic wavelength (267 nm), and the cumulative drug release was calculated against a standard calibration curve.

Under acidic conditions (pH 5.5), both the drug and the polymer become protonated and positively charged, which facilitates the release of Palbociclib due to electrostatic repulsion. After 12 h, the cumulative release reached 84.2%, indicating a substantial drug payload release in the acidic environment typical of tumor environment. In contrast, under physiological pH (7.4), the release rate was markedly slower, with only 17.87% of Palbociclib being released over the same period. This stark difference in release kinetics highlights the pH-responsive behavior of hPG-*b*-POX-Lys, suggesting that the zwitterionic and dendritic architecture of the polymer contributes to a controlled release mechanism sensitive to pH fluctuations (Figure 3). While the polymer system is designed to respond to the acidic tumor microenvironment, it is important to note that other cellular compartments, such as lysosomes and endosomes, also exhibit acidic pH values (typically pH 4.5–5.5). These intracellular acidic sites may influence the behavior of pH-responsive carriers and could potentially contribute to off-target drug release. Therefore, further studies are warranted to fully evaluate the impact of such acidic compartments on drug delivery and off-target effects.

**Figure 3:**
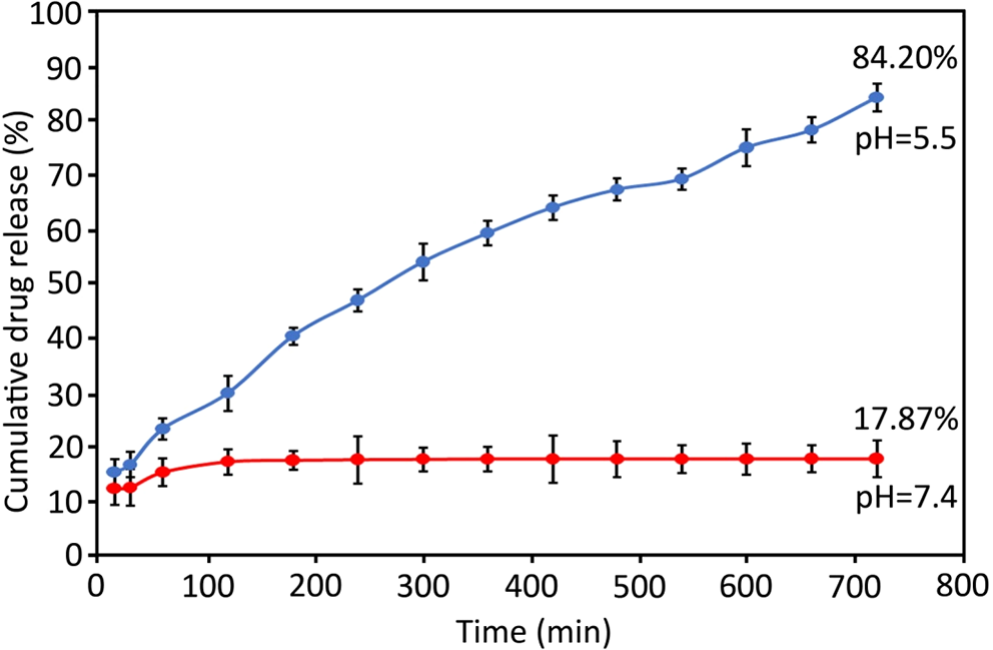
In vitro drug release profiles of hPG-*b*-POX-Lys at pH 7.4 and pH 5.5, demonstrating pH-dependent release kinetics.

These findings underscore the potential of hPG-*b*-POX-Lys as an intelligent drug delivery system capable of releasing its therapeutic payload preferentially in acidic tumor microenvironments, thereby minimizing systemic exposure and enhancing localized therapeutic action. This selective release not only enhances the efficacy of Palbociclib but also reduces the risk of off-target toxicity, which is crucial for improving patient outcomes in chemotherapeutic applications.

In vitro antibacterial assays demonstrated that the synthesized zwitterionic hPG-*b*-POX-Lys exhibited potent activity against both Gram-positive (*S. aureus* ATCC 25923) and Gram-negative (*E. coli* ATCC 25922) bacterial strains, while showing minimal cytotoxicity toward mammalian cells. The antibacterial efficacy was assessed using standard broth microdilution techniques according to CLSI guidelines,^44^ with an initial bacterial inoculum of 1 × 10^5^ CFU/mL in Mueller-Hinton broth to determine the minimum inhibitory concentration (MIC) and minimum bactericidal concentration (MBC).

A stock solution of hPG-*b*-POX-Lys (38 mg/mL) was serially diluted, and the results, summarized in Table S3, indicated MIC and MBC values as low as 0.5% (w/v) for both *S. aureus* and *E. coli*. Based on the stock concentration, this corresponds to an absolute antibacterial concentration of 190 μg/mL (0.005 × 38 mg/mL). The identical MIC and MBC values suggest that hPG-*b*-POX-Lys not only effectively inhibits bacterial growth but also eradicates the bacteria at the same concentration, highlighting its strong bactericidal properties rather than merely bacteriostatic effects.

In contrast, the regular hyperbranched polyglycerol (hPG) did not inhibit bacterial growth at any tested concentration up to 3%, indicating that the antibacterial activity is attributable to the POX-Lys functionalization rather than the hPG backbone (Figure S7). These findings underscore the potential of hPG-*b*-POX-Lys as a promising platform for the development of novel antibacterial agents, particularly in the context of rising antibiotic resistance.

The zwitterionic nature of hPG-*b*-POX-Lys is proposed to enhance its interaction with bacterial membranes through electrostatic attractions, thereby compromising membrane integrity. This effect is primarily observed in bacterial cells due to the distinct composition and higher density of negatively charged components in their membranes, whereas mammalian and cancer cell membranes exhibit lower surface charge, which explains the minimal cytotoxicity observed in human cells. This observation aligns with previously reported findings^24^ and is further supported by SEM images (Figure 5), which reveal pronounced morphological disruptions in treated bacterial cells, including cell wall disintegration and membrane rupture. Such interactions are critical for overcoming the defensive barriers of both Gram-positive and Gram-negative bacteria, highlighting hPG-*b*-POX-Lys as a promising candidate for combating multidrug-resistant pathogens. Despite being nonamphiphilic, this pseudodendrimer falls within the hyperbranched polymer family due to its dendritic architecture, controlled branching, and functional surface groups, which collectively contribute to its unique antibacterial properties.

The antibacterial efficacy of hPG-*b*-POX-Lys was further evaluated using the disk diffusion assay,^45^ Mueller-Hinton agar plates were inoculated with *S. aureus* and *E. coli*, and sterile filter disks impregnated with 190 μg/mL of hPG-*b*-POX-Lys were applied. Chloramphenicol served as a positive control due to its broad-spectrum activity, while polyglycerol (190 μg/mL) was used as a negative control to assess the baseline effect of the dendritic core material (Figure S8).

After 24 h of incubation at 37 °C, the zones of inhibition were measured. As summarized in Table S5, hPG-*b*-POX-Lys exhibited clear zones of inhibition of 16 mm for *E. coli* and 21 mm for *S. aureus*. In comparison, chloramphenicol showed 25 mm and 31 mm zones of inhibition, respectively, while polyglycerol had no measurable effect. These results confirm that the antibacterial activity arises from the POX-Lys functionalization, with the zwitterionic nature likely facilitating electrostatic interactions that destabilize bacterial membranes and lead to cell death. The difference in inhibition diameters between Gram-positive and Gram-negative bacteria reflects variations in cell wall composition, yet the polymer remains consistently effective.

To further validate antibacterial potency, a Bacterial Colony Count Assay was performed to assess the reduction in colony-forming units (CFUs) of *E. coli* and *S. aureus* over time. Cultures treated with hPG-*b*-POX-Lys were sampled at 1, 3, and 6-h intervals, and CFU counts (Table 1) revealed a substantial, time-dependent reduction, confirming the potent bactericidal activity of the polymer.

After just 1 h of exposure, hPG-*b*-POX-Lys achieved a 90% reduction in *E. coli* viability and a 50% reduction in *S. aureus*, corresponding to a logarithmic reduction (LR) of 1.0 and 0.301, respectively. Extending the exposure to 3 h enhanced bacterial killing, resulting in more than 99% reduction for both strains (LR > 2 for *E. coli* and 2 for *S. aureus*). By the 6-h mark, hPG-*b*-POX-Lys completely eradicated detectable CFUs of both *E. coli* and *S. aureus*, achieving over 99.9% reduction (LR > 5 for *E. coli* and >4 for *S. aureus*) (Figure 4, Table 2).

**Figure 4:**
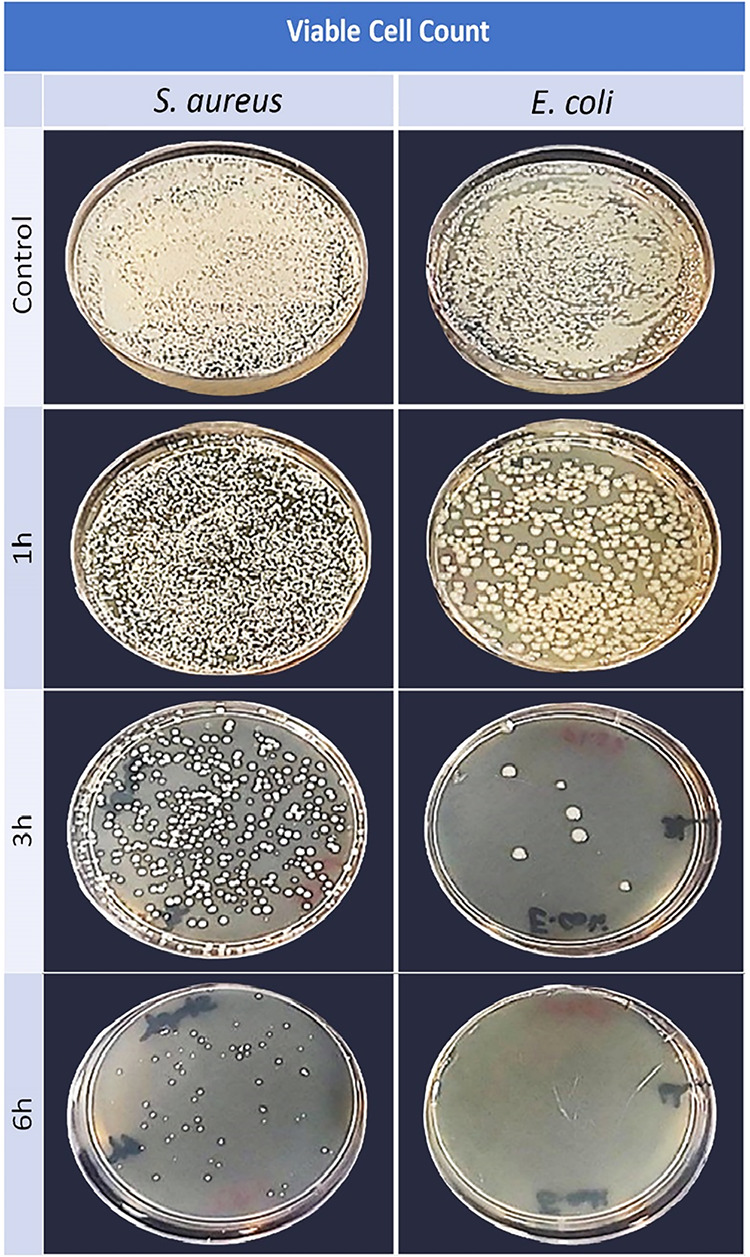
Representative images of hPG-*b*-POX-Lys-treated samples and colonies in culture medium at 1, 3, and 6 h.

**Table 2:** Bacterial Colony Counts Assay Results of hPG-*b*-POX-Lys

Strain: *E. coli*									
Sample	1 h			3 h			6 h		
	VC^1^ (CFU/mL)	RP^2g^ (%)	LR^3^ (Log_10_)	VC^1^ (CFU/mL)	RP^2^(%)	LR^3^ (Log_10_)	VC^1^ (CFU/mL)	RP^2^ (%)	LR^3^ (Log_10_)
A3	1 × 10^5^	90%	1	<1 × 10^3^	>99%	>2	<1 × 10^1^	>99.9%	>5
1- Viable Count, 2- Reduction Percentage, 3- Logarithmic Reduction									

Strain: *S. aureus*									
Sample	1 h			3 h			6 h		
	VC^1^ (CFU/mL)	RP^2^ (%)	LR^3^ (Log_10_)	VC^1^ (CFU/mL)	RP^2^ (%)	LR^3^ (Log_10_)	VC^1^ (CFU/mL)	RP^2^ (%)	LR3 (Log_10_)
A3	5 × 10^5^	50%	0.301	1 × 104	>99%	2	<1 × 10^2^	>99.9%	>4
1-Logarithmic Reduction, 2-Reduction Percentage, 3-Viable Count									

These results underscore the rapid and potent antibacterial activity of hPG-*b*-POX-Lys in a time-dependent manner, likely mediated by membrane destabilization and interference with essential bacterial processes. The complete bactericidal effect within 6 h highlights the potential of hPG-*b*-POX-Lys as a robust antimicrobial agent for combating both Gram-negative and Gram-positive bacteria.

The effect of hPG-*b*-POX-Lys on bacterial morphology was visualized using Scanning Electron Microscopy (SEM), providing direct evidence of its bactericidal mechanism. SEM images revealed substantial structural damage in both *S. aureus* and *E. coli* upon exposure to the polymer. Treated bacterial cells exhibited severe membrane disruptions, deep surface cracks, and pronounced surface depressions, all indicative of compromised cell wall integrity. In contrast, untreated controls maintained smooth, intact cellular morphologies without visible damage (Figure 5).

**Figure 5:**
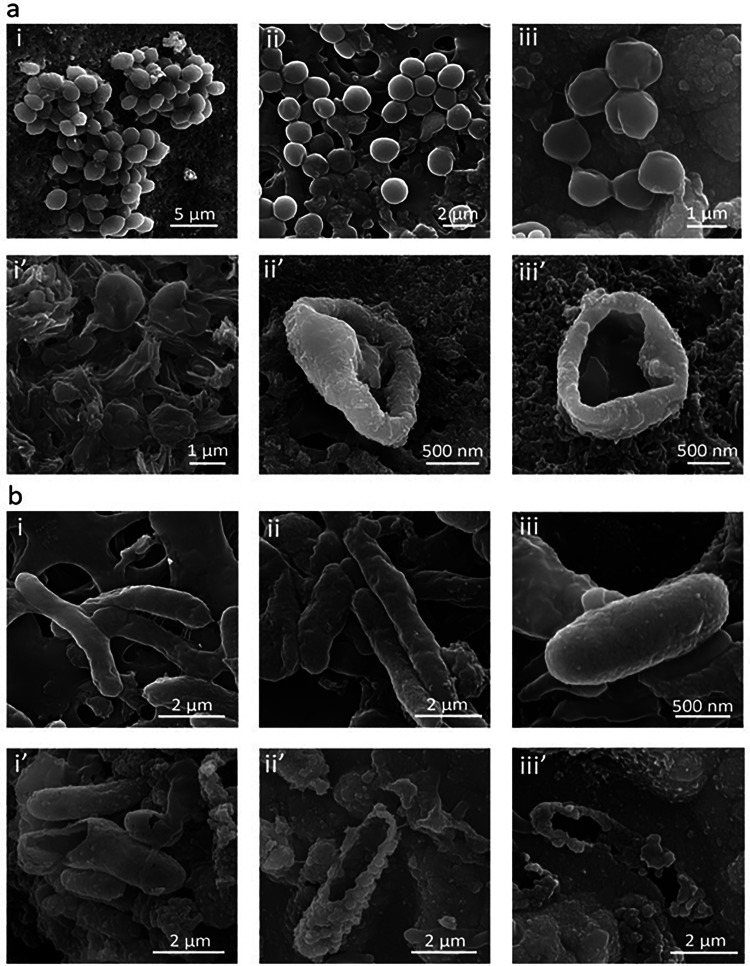
SEM images of (a) *S. aureus* and (b) *E. coli* bacteria (i–iii) before and (i’–iii’) after exposure to the hPG-*b*-POX-Lys pseudodendrimer.

These pronounced morphological alterations suggest that hPG-*b*-POX-Lys interacts electrostatically with the negatively charged bacterial cell membrane.^46^ The zwitterionic nature of the dendrimer likely enhances its affinity for the bacterial surface, leading to membrane destabilization. This disruption increases membrane permeability, causes leakage of intracellular contents, and ultimately results in bacterial cell lysis.

The dual functionality of this platform, namely its ability to load anticancer drugs and its intrinsic antibacterial activity, suggests potential applications in therapeutic contexts where bacterial infection and cancer coexist or where infection risk is elevated during chemotherapy, such as in tumor-associated infections, postsurgical cancer treatments, or immunocompromised patients undergoing chemotherapy. In such cases, a multifunctional system capable of simultaneous antibacterial protection and anticancer drug delivery could provide important therapeutic benefits.^47,48^

Although the overall macromolecular architecture of the present polymer is related to our previously reported system,^40^ the substitution of arginine with lysine residues at the polymer termini introduces several important physicochemical and biological differences. These differences primarily arise from the distinct side-chain chemistries of the two amino acids. Arginine contains a guanidinium group with a very high p*K*_a_ (∼12.5), which remains strongly positively charged over a broad pH range. This permanent cationic character promotes strong electrostatic interactions with negatively charged biomolecules and cellular membranes. In contrast, lysine contains a primary amine group (p*K*_a_ ∼ 10.5) that is more sensitive to environmental conditions and can participate in dynamic protonation–deprotonation equilibria.

As a consequence of these differences, the lysine-functionalized polymer described in this work exhibits a distinct charge regulation behavior. In our system, the combination of lysine residues and the polymer backbone creates a zwitterionic-like balance, resulting in an overall near-zero net charge across a broad pH window (approximately pH 4–9). This behavior contrasts with the arginine-containing analogue, which maintains a strongly positive surface charge over the same pH range. Such a charge profile can significantly influence interactions with biological systems. In particular, the near-neutral charge state over physiologically relevant pH conditions may reduce nonspecific interactions with mammalian cell membranes while still enabling effective interactions with bacterial membranes. This characteristic likely contributes to the favorable biological performance of hPG-*b*-POX-Lys, including its high antibacterial activity and promising anticancer properties observed in the present study.

## Conclusion

In this study, the pseudodendrimer polyglycerol–polyoxazoline–lysine (hPG-*b*-POX-Lys) was successfully synthesized on a gram scale. The resulting polymer exhibited notable antibacterial activity against *S. aureus* and *E. coli*, with minimum inhibitory concentration (MIC) and minimum bactericidal concentration (MBC) values of 0.19 mg/mL.

Cytotoxicity evaluation demonstrated a favorable safety profile of the carrier polymer, with no significant reduction in viability of MCF-7, HeLa, and human fibroblast cells within the tested concentration range. The hPG-*b*-POX-Lys+Pal complex showed enhanced antiproliferative activity compared with free Palbociclib, with IC_50_ values decreasing from 5.013 to 3.48 μg/mL in MCF-7 cells and from 11.69 to 7.57 μg/mL in HeLa cells. In contrast, neither the carrier nor the hPG-*b*-POX-Lys+Pal complex reached IC_50_ in human fibroblasts within the studied concentrations, indicating reduced cytotoxicity toward nonmalignant cells.

Drug loading studies revealed a high loading efficiency of 86%, and in vitro release experiments confirmed a pH-responsive release behavior, with 84.2% of Palbociclib released at pH 5.5 compared to 17.87% at pH 7.4, supporting preferential drug release under acidic conditions.

Overall, hPG-*b*-POX-Lys represents a multifunctional platform combining antibacterial activity with efficient and selective anticancer drug delivery. Its pH-triggered release, high loading capacity, and differential cytotoxicity profile make it a promising candidate for dual antibacterial and tumor-targeted therapeutic applications, while further in vivo studies are required to refine the therapeutic window and translational potential.

## Supplementary Material



## Notes

Detailed experimental procedures (materials and methods), synthetic protocols, and comprehensive characterization data, including spectroscopic and microscopic analyses. Additional information on drug loading efficiency, in vitro toxicity studies, and antibacterial activity assays is also provided (PDF)
