# Supplementary material for: Circular-by-Design Zwitterionic Polyglycerol–Polyoxazoline–Lysine Pseudodendrimers as Multifunctional Antibacterial and Anticancer Drug Delivery Platforms
Source: Biomacromolecules. 2026 May 7;27(7):4474–85. doi: 10.1021/acs.biomac.6c00382 (PMC13370777; doi:10.1021/acs.biomac.6c00382)
Supplement: Supplementary file 1 [file bm6c00382_si_001.pdf]

Supporting Information

Circular-by-Design Zwitterionic Polyglycerol-Polyoxazoline-Lysine Pseudodendrimers as Multifunctional Antibacterial and Anticancer Drug Delivery Platforms

Behnaz Bastami[a], Parviz Rashidi Ranjbar[b], Mohsen Adeli[c,d]*, Siamak Beyranvand[c]*

[a] Kish International Campus, University of Tehran, Kish Island 39982-79416, Iran,

[b]School of Chemistry, College of Science, University of Tehran, Tehran 141556455, Iran,

[c]Department of Organic Chemistry, Faculty of Chemistry, Lorestan University, Khorramabad 68141-54316, Iran

[d] Institut für Chemie und Biochemie, Freie Universität Berlin, Takustr. 3, 14195 Berlin, Germany

Corresponding authors:

M.A: adeli.m@lu.ac.ir, m.aadeli@fu-berlin.de

P.R.R: parvizrashidi2@ut.ac.ir

Table of Contents

Experimental....................2

Materials....................2

Methods....................2

Synthesis of mesylated hyperbranched polyglycerol (hPG-OMS).........**Error! Bookmark not defined.**

Synthesis of Poly(glycerol-*b*-2-ethyl-2-oxazoline)-lysine (hPG-*b*-POX-Lys)**Error! Bookmark not defined.**

Zone of Inhibition Assay (Well Diffusion Method)....................**Error! Bookmark not defined.**

Cell Viability Assessment Using the MTT Assay....................**Error! Bookmark not defined.**

Preparation of Palbociclib-Loaded hPG-*b*-POX-Lys (hPG-*b*-POX-Lys+Pal.)**Error! Bookmark not defined.**

Determination of Minimum Inhibitory Concentration (MIC) and Minimum Bactericidal Concentration (MBC)....................**Error! Bookmark not defined.**

Determination of Drug Loading Capacity (LC) and Drug Loading Efficiency (DLE)....................8

References....................**Error! Bookmark not defined.**

Experimental

Materials

Mesyl chloride (CH_3SO_2Cl), 2-ethyl-2-oxazoline, triethylamine (Et_3N), acetonitril (CH_3CN), acetone ($(CH_3)_2CO$), pyridine (C_5H_5N), ethanol (C_2H_5OH), dimethylformamide (DMF) and dimethylsulfoxide (DMSO) were purchased from Merck. L-lysine, 3-(4,5-Dimethylthiazol-2-yl)-2,5-Diphenyltetrazoliumbromid, EDTA (ethylene diamine tetraacetic acid), and trypan blue were purchased from Sigma Aldrich. Trypsin and DMEM (Dulbecco's Modified Eagle Medium) were purchased from bioidea- made in Iran. Hyperbranced polyglycerol (hPG) (M_n = 5000 g/mol) was synthesized using anionic ring opening polymerization of glycidol by Prof. Dr. Rainer Haag group. Regenerated cellulose (RC) membrane filters (width: 31 mm, MWCO = 2 kDa) were purchased from Fischer Scientific (Geel -Belgium). *Escherichia coli* strain (*E. coli*) (ATCC 25922), *Staphylococus aureus* ATCC 25923, MCF7 and Hela cell lines were supplied from the Pasteur Institute, Tehran- Iran.

Methods

Fourier-transform infrared (FTIR) spectroscopy was conducted using a Shimadzu IR 8400 spectrometer. The samples were prepared using the KBr pellet technique, and spectra were recorded in the 4000 to 500 cm^{-1} range with a sample-to-KBr weight ratio of 5 mg to 200 mg. The Nuclear magnetic resonance (NMR) spectra were performed on a Brucker AMX 500 spectrometer. Inverse-gated ^{13}C NMR was performed on Brucker Avance 400. Scanning electron microscopy (SEM) images were captured using a TESCAN instrument from Brno, Czech Republic, to evaluate the surface morphology of the materials. The optical properties of the samples were analyzed through UV-Vis spectroscopy using a Shimadzu UV-Vis 1650 PC spectrophotometer equipped with a 1.0 cm quartz cell. Thermal stability and transitions were assessed through thermogravimetric analysis (TGA) and differential scanning calorimetry (DSC) using TGA 1 and DSC 1 models from Mettler Toledo, Switzerland, respectively. To determine particle size distribution, dynamic light scattering (DLS) analysis was performed using a

Malvern ZN Series instrument. This method provided size measurements within the range of 1 to 6000 nanometers, adhering to ISO 3001 standards. Additionally, zeta potential measurements were carried out using the same instrument, with a measurement range of +1000 to -1000 millivolts, following ISO 3002 standards. Further characterization of the synthesized compounds was achieved using transmission electron microscopy (TEM) with a Philips TEM CM120 model to examine the internal structure at high resolution. Finally, elemental analysis (CHNS) was performed using an Eager 300 model for EA1112 to determine the composition of carbon, hydrogen, nitrogen, and sulfur in the synthesized compounds. This comprehensive array of materials and methods ensured the reliable synthesis, characterization, and analysis of the novel pseudo-dendrimer, facilitating a thorough investigation of its properties and applications. The images were taken by a Smartphone camera (48 megapixels) under variable lighting conditions.

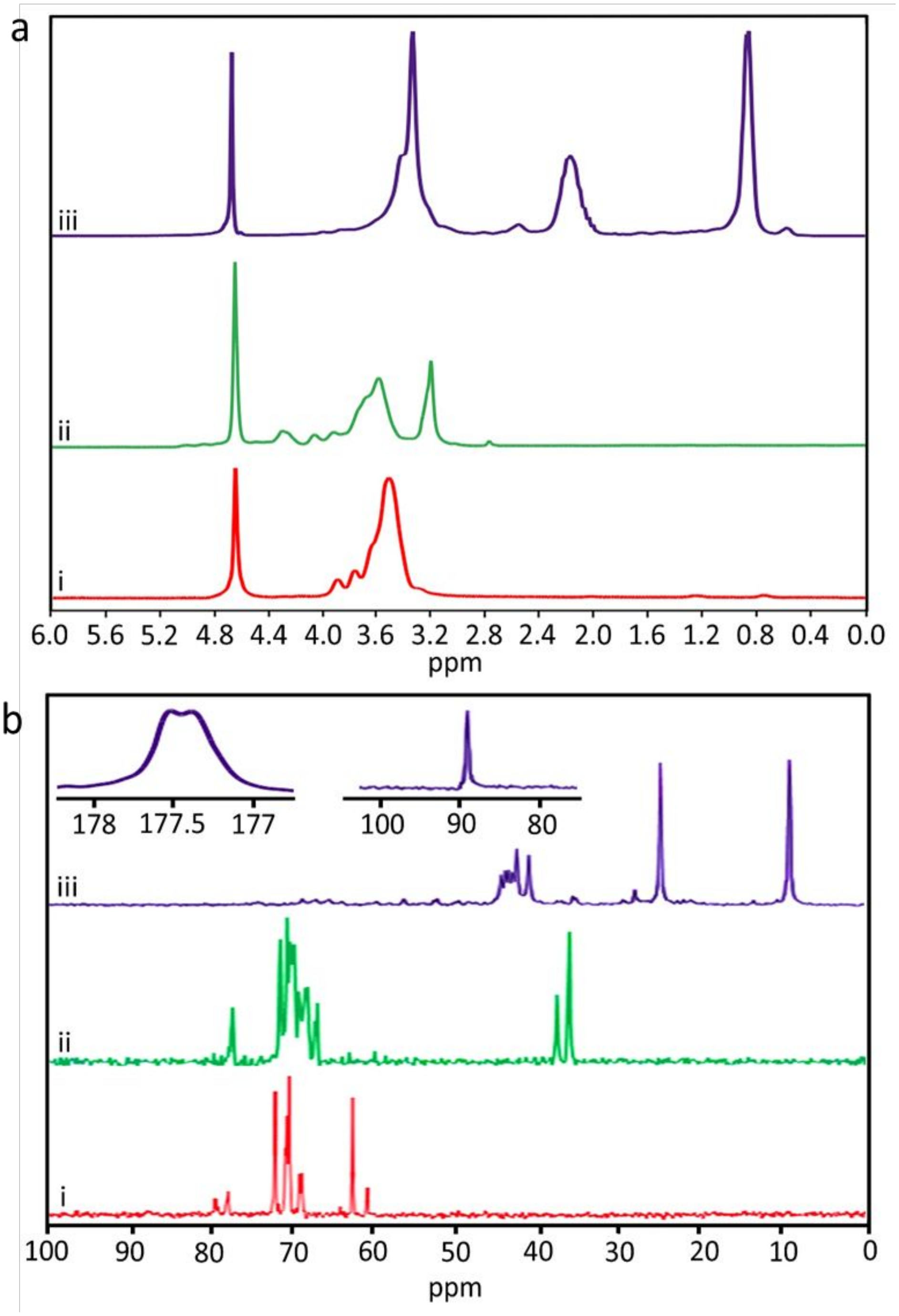

Figure S1. Expanded a)^{1}HNMR and b)^{13}CNMR spectra of i) hPG, ii) hPG-OMS, and iii) hPG-*b*-POX-Lys.

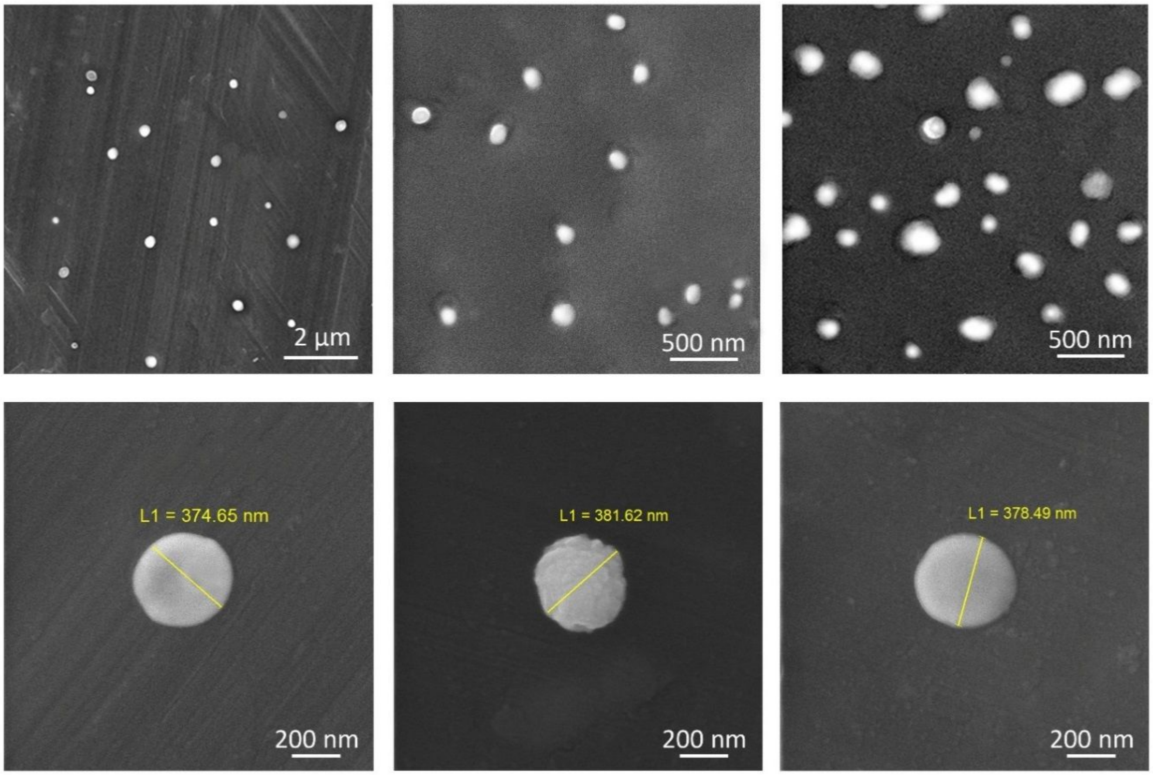

Figure S2. SEM images of hPG-*b*-POX-Lys.

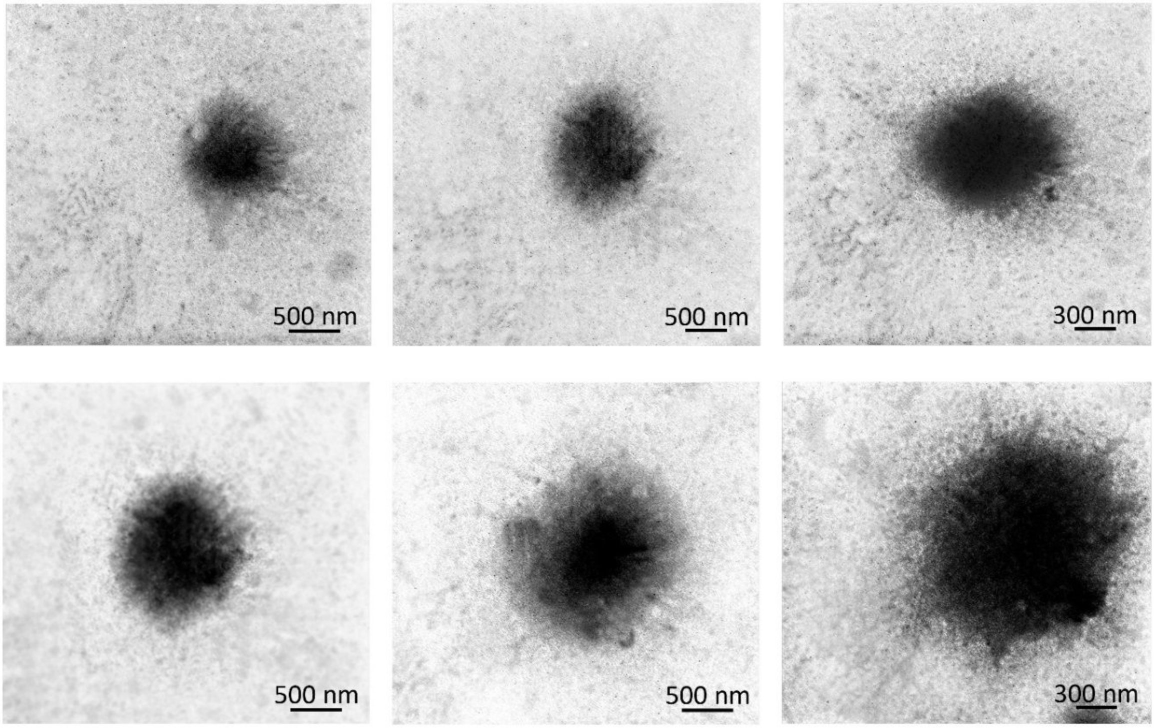

Figure S3. TEM images from hPG-*b*-POX-Lys.

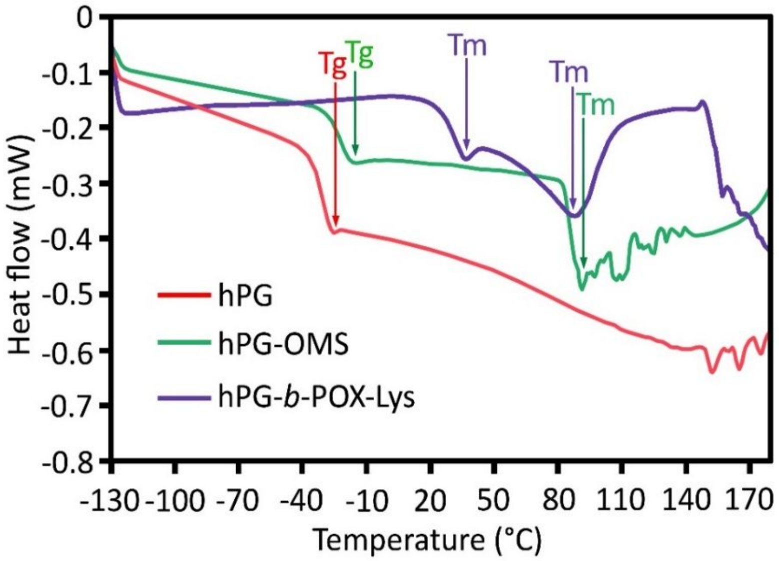

Figure S4. DSC diagram of hPG, hPG-OMS, hPG-*b*-POX-Lys.

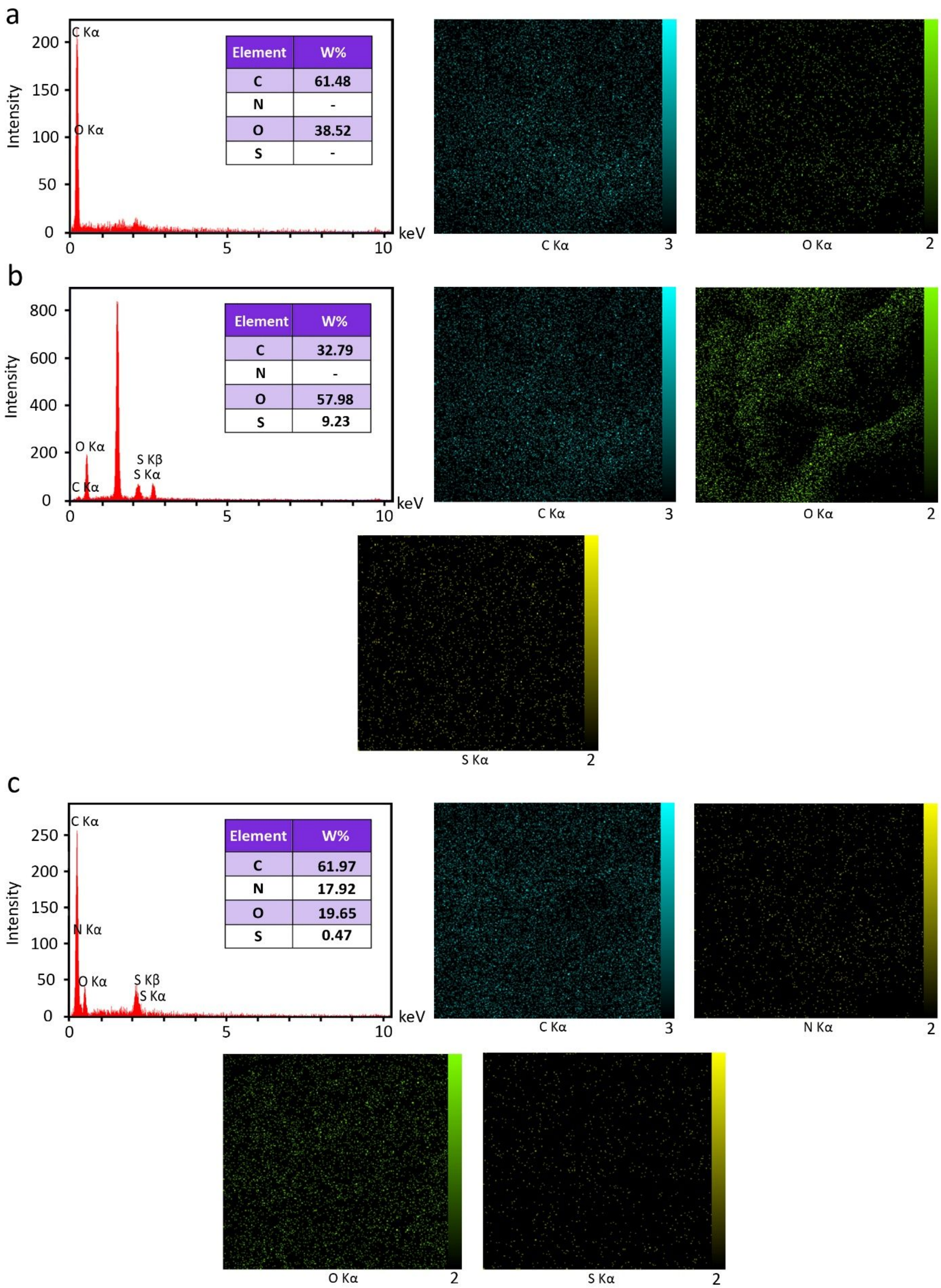

Figure S5. EDAX analysis of hPG, hPG-OMS, hPG-*b*-POX-Lys.

Determination of Drug Loading Capacity (LC) and Drug Loading Efficiency (DLE)

Drug incorporation parameters for the hPG-*b*-POX-Lys pseudo-dendrimer were quantified using two standard metrics: loading capacity (LC) and drug loading efficiency (DLE).

Loading capacity (LC) represents the fraction of the final drug-loaded formulation that corresponds to the mass of the incorporated drug and was calculated using the following equation:

$$\mathrm{LC}\ (\%) = \frac{\text{Weight of the encapsulated drug}}{\text{Weight of the copolymer} + \text{Weight of the encapsulated drug}} \times 100$$

Drug loading efficiency (DLE), also referred to as encapsulation efficiency, represents the fraction of the initially added drug that was successfully incorporated into the carrier:

DLE (%) = (Weight of encapsulated drug / Weight of drug initially added) × 100

To determine the amount of incorporated Palbociclib, the dried polymer–drug complex was dissolved in PBS and analyzed by UV–Vis spectroscopy at 267 nm. Quantification was performed using a previously established calibration curve:

$y = 0.0176x - 0.0882$ ($R^2 = 0.9966$)

where *y* is the measured absorbance and *x* is the drug concentration (μg/mL).

All measurements were performed in triplicate, and the average value was used for LC and DLE calculations. Based on UV–Vis quantification, the drug loading efficiency (DLE) was determined to be 86%, while the loading capacity (LC) reached 81.25%, indicating efficient incorporation and a high payload fraction of Palbociclib within the polymer carrier.

Table S1. Determination of The LC of hPG-*b*-POX-Lys.

Sample	Total drug used (mg)	Loaded drug (mg)	Loading Capacity (%)
**hPG-*b*-POX-Lys**	0.15 mg	0.13 mg	81.25 %

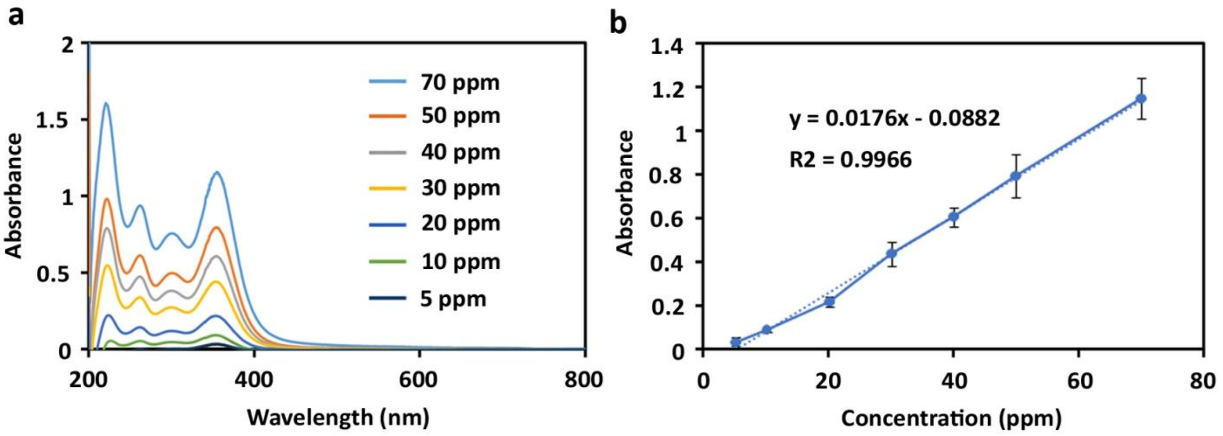

Figure S6. UV–vis calibration curve of Palbociclib recorded at 267 nm with the associated linear regression parameters.

Figure S7 presents the concentration-dependent effects of free Palbociclib, the hPG-*b*-POX-Lys+Pal. complex, and the blank hPG-*b*-POX-Lys carrier on cell viability after 48 h incubation as measured by the MTT assay. In MCF-7 and HeLa cancer cell lines (Figure S7a), both free Palbociclib and the polymer–drug complex produced a clear dose-dependent reduction in cell viability across the tested concentration range. The hPG-*b*-POX-Lys+Pal. complex consistently induced a stronger decrease in viability than the free drug at equivalent concentrations, particularly in the mid-dose region, indicating enhanced antiproliferative activity when Palbociclib is delivered via the polymeric carrier. Meanwhile, in human fibroblasts, the hPG-b-POX-Lys+Pal complex exhibited low cytotoxicity, maintaining favorable cell viability throughout the tested concentration range, thereby demonstrating its good cytocompatibility in normal cells. In contrast, treatment with the blank hPG-*b*-POX-Lys polymer alone (Figure S7b) resulted in minimal reduction of viability in MCF-7, HeLa, and human fibroblast cells, with cell survival remaining high even at elevated concentrations. Only a modest decline in viability was observed at the highest tested doses, supporting the low intrinsic cytotoxicity and good biocompatibility of the carrier. Together, these results demonstrate that drug loading into hPG-*b*-

POX-Lys enhances anticancer efficacy while the unloaded polymer exhibits limited toxicity toward both malignant and non-malignant cells.

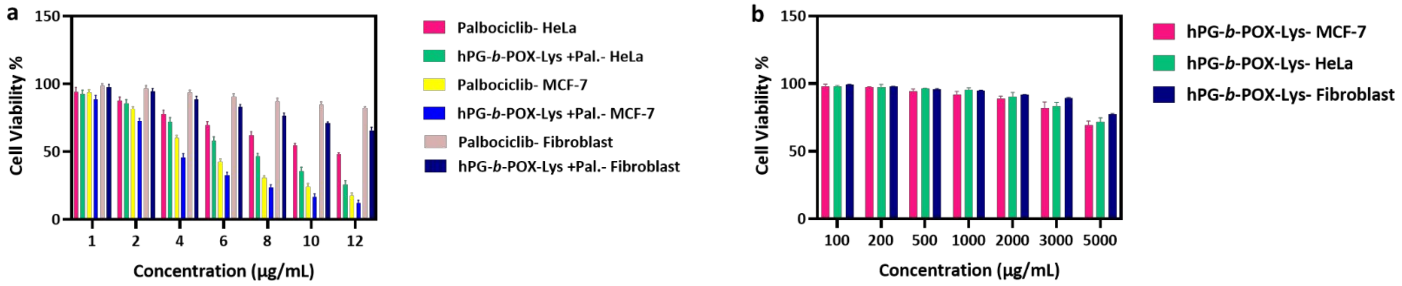

Figure S7. Concentration-dependent cell viability of cancer and normal cells after treatment with free Palbociclib, hPG-*b*-POX-Lys+Pal. complex, and blank hPG-*b*-POX-Lys polymer for 48 h, determined by the MTT assay. (a) Viability of MCF-7 , HeLa and human fibroblast cells treated with free Palbociclib and hPG-*b*-POX-Lys+Pal. across the indicated concentration range, showing a dose-dependent reduction and enhanced antiproliferative effect for the polymer–drug complex. (b) Viability of MCF-7, HeLa, and human fibroblast cells treated with hPG-*b*-POX-Lys alone, demonstrating high cell survival over a wide concentration range and low intrinsic cytotoxicity of the carrier. Data are presented as mean ± SD (n = 3).

Table S2. MIC and MBC values of hPG-***b***-POX-Lys against gram-positive and gram-negative bacteria**.**

**hPG-*b*-POX-Lys**	**MIC**	**MBC**
S. aureus	190 µg/mL	190 µg/mL
E. coli	190 µg/mL	190 µg/mL

Table S3. Disk diffusion assay results for hPG-*b*-POX-Lys and controls.

**Concentration**	**Sample**
**Strain**	***E. coli***
**Diameter of zone of inhibition hPG-*b*-POX-Lys 0.5%**	16 mm
**Diameter of zone of inhibition of control (chloramphenicol)**	25 mm
**Strain**	***S. aureus***

**Diameter of zone of inhibition hPG-*b*-POX-Lys 0.5%**	21 mm
**Diameter of zone of inhibition of control (chloramphenicol)**	31 mm

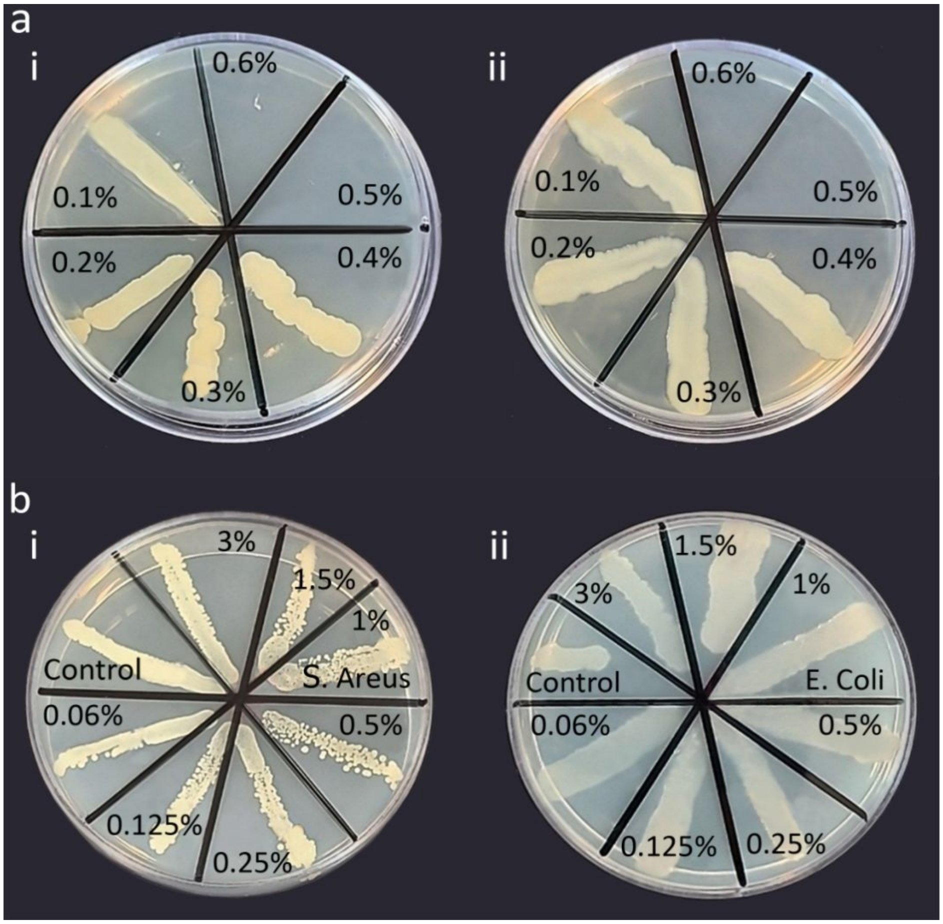

Figure S8. MBC determination of a) hPG-*b*-POX-Lys and b) hPG against i) *S. aureus* and ii) *E. coli*, illustrating complete bacterial clearance at 190 µg/mL concentration.

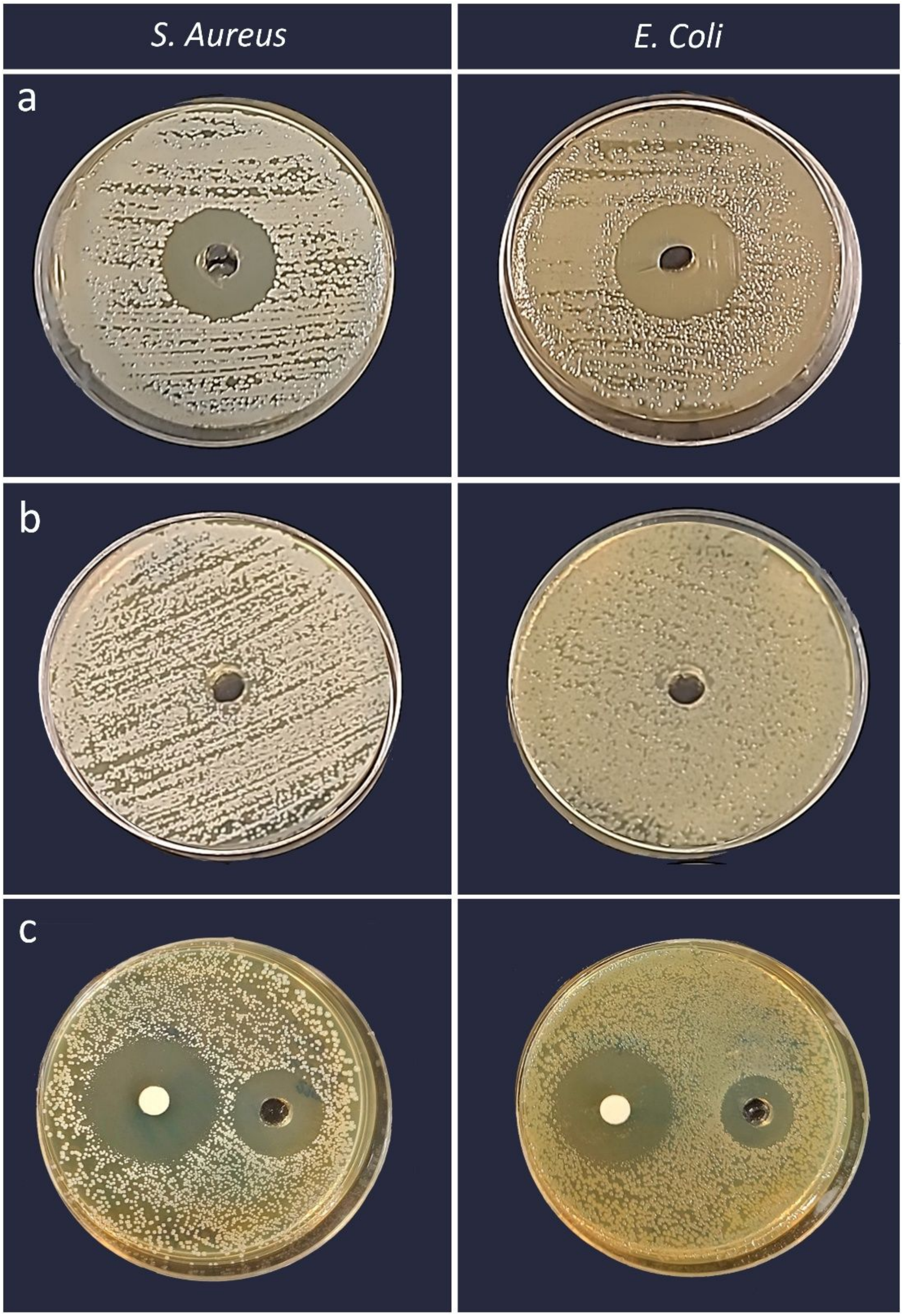

Figure S9. Disk diffusion test results: a) hPG-*b*-POX-Lys, b) polyglycerol (negative control), and) c) chloramphenicol (positive control) against gram-positive and gram-negative bacteria.
